# Supplementary material for: Protein Fortification of Millet-Based Gluten-Free Snacks Designed for 3D Printing
Source: Foods. 2025 Dec 14;14(24):4308. doi: 10.3390/foods14244308 (PMC12733089; doi:10.3390/foods14244308)
Supplement: Supplementary file 1 [file foods-14-04308-s001.zip › foods-4025214-supplementary.pdf]

Supplementary Table S1. Effect of protein content on shape fidelity, deformation, and hardness of 3D-printed millet-based snacks.

	Shape fidelity (%)			Deformation (%)			Texture	Appearance	
	Length	Width	Height	Length	Width	Height	Hardness (N)	Top view	Lateral view
**Control snack**	105.30 ±0.65	109.01 ±1.45	77.78 ±3.55	3.64 ±0.29	3.75 ±0.73	14.02 ±1.86	12.13±0.52	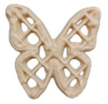	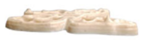
**12% protein**	104.54 ±0.74	106.41 ±0.38	90.50 ±1.43	4.69 ±0.48	2.74 ±0.37	11.02 ±2.26	13.24±1.05	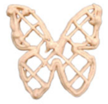	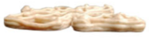
**16% protein**	103.29 ±0.73	105.06 ±0.62	96.83 ±0.88	4.34 ±0.89	2.31 ±0.31	2.98 ±0.42	15.22±0.77	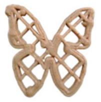	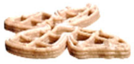
**20% protein**	98.84 ±0.44	93.07 ±0.60	94.29 ±1.24	6.56 ±0.16	4.17 ±0.37	3.91 ±0.04	19.84±0.64	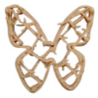	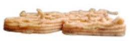

Supplementary Table S2. One-way ANOVA and Tukey HSD (Control vs 12%, 16%, 20%).

Outcome	ANOVA F(df), p-value	Pairwise (Tukey HSD, group1 – group2)	Mean diff	Significant (α = 0.05)
Shape fidelity	$F(3,8) = 67.19$, $p < 0.001$	12% – 16%	−1.244	No
		12% – 20%	5.083	Yes
		12% – Control	3.122	Yes
		16% – 20%	6.328	Yes
		16% – Control	4.367	Yes
		20% – Control	−1.961	Yes
Deformation	$F(3,8) = 35.64$, $p < 0.001$	12% – 16%	−2.941	Yes
		12% – 20%	−1.650	No
		12% – Control	0.987	No
		16% – 20%	1.669	Yes
		16% – Control	3.928	Yes
		20% – Control	2.259	Yes
Hardness	$F(3,8) = 41.19$, $p < 0.001$	12% – 16%	1.987	No
		12% – 20%	6.603	Yes
		12% – Control	−1.107	No
		16% – 20%	4.617	Yes
		16% – Control	−3.093	Yes
		20% – Control	−7.710	Yes

Supplementary Table S3. Effect of protein ratio on shape fidelity, deformation, and hardness of 3D-printed millet-based snacks.

	Shape fidelity (%)			Deformation (%)			Texture	Appearance	
	Length	Width	Height	Length	Width	Height	Hardness (N)	Top view	Lateral view
**Control snack**	105.30 ±0.65	109.01 ±1.45	77.78 ±3.55	3.64 ±0.29	3.75 ±0.73	14.02 ±1.86	12.13±0.52	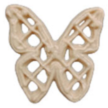	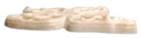
**100:0**	103.71 ±0.13	104.13 ±0.20	96.44 ±2.55	5.38 ±0.05	3.69 ±0.82	8.82 ±1.64	24.03±0.26	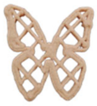	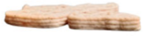
**75:25**	104.46 ±0.33	105.22 ±0.12	96.61 ±1.17	5.65 ±0.77	3.34 ±0.26	11.03 ±1.74	21.71±0.79	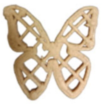	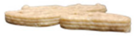
**50:50**	103.29 ±0.73	105.06 ±0.62	96.83 ±0.88	4.34 ±0.89	2.31 ±0.31	2.98 ±0.42	15.22±0.77	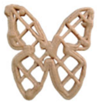	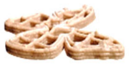
**25:75**	104.18 ±0.36	105.91 ±0.66	95.33 ±1.43	5.22 ±0.38	3.34 ±0.52	7.44 ±1.36	7.48±0.83	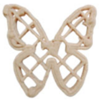	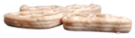
**0:100**	104.57 ±0.91	105.33 ±0.66	92.50 ±2.68	5.44 ±0.17	3.26 ±0.18	7.26 ±1.31	3.58±0.43	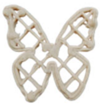	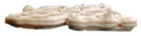

Supplementary Table S4. One-way ANOVA and Tukey HSD (almond:yeast protein ratio at 16% total protein: 100:0, 75:25, 50:50, 25:75, 0:100).

Outcome	ANOVA F(df), p-value	Pairwise (Tukey HSD, group1 – group2)	Significant (α = 0.05)
Shape fidelity	F(4,10) = 0.506, p = 0.733	All pairwise contrasts	No
Deformation	F(4,10) ≈ 12.84, p < 0.001	50:50 vs 100:0	Yes
		50:50 vs 0:100	Yes
		50:50 vs 75:25	Yes
		50:50 vs 25:75	Yes
Hardness	F(4,10) ≈ 362.17, p ≪ 0.001	100:0 vs 0:100	Yes
		100:0 vs 50:50	Yes
		100:0 vs 75:25	Yes
		100:0 vs 25:75	Yes

Consumer Study on 3D Printed Snacks – Participant Information

Nutrition and health are closely connected, and consumers today are increasingly demanding healthier and more innovative food products. Within this study, we are developing 3D printed snack products designed to be nutritionally valuable, visually appealing, and tailored to consumer needs.

Our goal is to better understand your attitudes and preferences regarding these novel products. Your feedback will help us improve the recipes and adjust them to consumer expectations.

The study is conducted by the Institute of Food Technology (FINS) and the BioSense Institute in Novi Sad. Participation is voluntary and anonymous, and all responses will remain strictly confidential and used only for research purposes. You may withdraw at any time without explanation.

The research protocol has been approved by the Ethics Committee of the Institute of Food Technology in Novi Sad (Approval No. 24-58-3, 16.12.2024).

Completing the questionnaire will take about 15 minutes.

We truly appreciate your time and valuable contribution!

This study is part of the project "Cereal-based 3D printed snack with plant proteins – REPRINT3D," funded by FINS and supported through the SAIGE program (Republic of Serbia, Ministry of Science, World Bank, and European Union).

SECTION 1: DEMOGRAPHIC DATA
Check the appropriate box

Gender?

☐ *Male*
☐ *Female*

Age?

☐ *18-24*
☐ *25-34*
☐ *35-44*
☐ *45-54*
☐ *55-65*
☐ *65+*

Education?

☐ Primary school
☐ High school
☐ College / Vocational school
☐ University / Master's degree
☐ PhD

Employment status?

☐ *Student*
☐ *Employed part-time*
☐ *Employed full-time*

Monthly income

☐ *<400€*
☐ *400-600€*
☐ *600-800€*
☐ *800-1000€*
☐ *>1000€*

Consumer Test – Evaluation Sheet

Instructions:

You have two samples in front of you. The samples are labeled with numbers. Please:

- Taste the samples one by one.
- Do not open all the bags at once; instead, open them one at a time as you evaluate the products.
- When evaluating, take short breaks between samples, drink some water, and allow your senses to rest.

SAMPLE NUMBER 1

Please evaluate the following product characteristics:
Rate on a scale from **1 to 7**, where **1 = Dislike very much** and **7 = Like very much**.

1. How much do you like the **appearance** of the product?

- Sample 1: [1-2-3-4-5-6-7]

2. How much do you like the **flavor** of the product?

- Sample 1: [1-2-3-4-5-6-7]

3. How much do you like the **texture** of the product?

- Sample 1: [1-2-3-4-5-6-7]

4. How much do you like the **color** of the product?

- Sample 1: [1-2-3-4-5-6-7]

5. How much do you like the **aroma** of the product?

- Sample 1: [1-2-3-4-5-6-7]

6. How much do you like the **crispness** of the product?

- Sample 1: [1-2-3-4-5-6-7]

. How much do you like the **smell** of the product?

- Sample 1: [1-2-3-4-5-6-7]

SAMPLE NUMBER 2

Please evaluate the following product characteristics:
Rate on a scale from **1 to 7**, where **1 = Dislike very much** and **7 = Like very much**.

2. How much do you like the **appearance** of the product?

- Sample 2: [1-2-3-4-5-6-7]

2. How much do you like the **flavor** of the product?

- Sample 2: [1-2-3-4-5-6-7]

3. How much do you like the **texture** of the product?

- Sample 2: [1-2-3-4-5-6-7]

4. How much do you like the **color** of the product?

- Sample 2: [1-2-3-4-5-6-7]

5. How much do you like the **aroma** of the product?

- Sample 2: [1-2-3-4-5-6-7]

6. How much do you like the **crispness** of the product?

- Sample 2: [1-2-3-4-5-6-7]

. How much do you like the **smell** of the product?

- Sample 2: [1-2-3-4-5-6-7]

Overall Evaluation

Please rate each sample overall, taking into account its appearance, texture, taste, and aroma.

- Sample 1: ☐ 1 ☐ 2 ☐ 3 ☐ 4 ☐ 5
- Sample 2: ☐ 1 ☐ 2 ☐ 3 ☐ 4 ☐ 5

Re-consumption

Would you be willing to consume this product again?

1. Sample 1: ☐ Yes ☐ No
2. Sample 2: ☐ Yes ☐ No
